# Acute myeloid leukemia presenting with gingival enlargement masked by a normal complete blood count: A case of delayed diagnosis and literature review

**DOI:** 10.1097/MD.0000000000046684

**Published:** 2025-12-19

**Authors:** Dandan Wu, Wenxiu Li, Liang Luo, Fugen Ding

**Affiliations:** a Department of Periodontology, Stomatological Hospital of Guizhou Medical University, Guiyang, China; b Department of General Dentistry, Stomatological Hospital of Guizhou Medical University, Guiyang, China.

**Keywords:** acute myeloid leukemia, dentistry, diagnosis, gingival enlargement, oral manifestations, oral medicine

## Abstract

**Rationale:**

Acute myeloid leukaemia (AML) is a lethal hematological malignancy whose oral manifestations are frequently misdiagnosed as inflammatory periodontal disease. This case exemplifies the diagnostic challenges associated with AML, particularly when initial blood counts appear normal and conventional periodontal treatment proves ineffective.

**Patient concerns:**

A 45-year-old male patient presented with a 3-month history of progressive gingival bleeding, swelling, and occlusal discomfort.

**Diagnoses:**

The preliminary hematological analysis yielded no significant findings. Not with standing the implementation of conventional periodontal therapy, the gingival enlargement exhibited a marked exacerbation, manifesting as a soft, lobulated appearance. A subsequent hematological workup revealed thrombocytopenia (68 × 10^9^/L). Bone marrow aspiration confirmed the diagnosis of AML with 81% myeloblasts.

**Interventions:**

The patient received periodontal initial therapy and “7 + 3” induction chemotherapy regimen (cytarabine + daunorubicin).

**Outcomes:**

The chemotherapy resulted in significant regression of the gingival enlargement. However, the patient passed away 5 months after diagnosis.

**Lessons:**

This case underscores the notion that gingival enlargement can serve as an initial indicator of AML, even in the absence of conventional hematological abnormalities. Dentists are required to exercise a high level of suspicion regarding systemic etiologies when confronted with severe, refractory, or rapidly progressive gingival enlargement that is disproportionate to local irritants. It is imperative that patients consult with hematologists at the earliest opportunity, and that repeated hematological evaluations are carried out in order to prevent misdiagnosis and delays in life-saving treatment.

## 1. Introduction

Acute myeloid leukemia (AML) is a hematological malignancy characterized by the clonal expansion of myeloid blasts in the peripheral blood, bone marrow, and other tissues. It is the predominant form of acute leukemia in adults and is known for its significant heterogeneity.^[1]^ AML causes more than 80,000 deaths worldwide annually, and this number is projected to double within the next 2 decades.^[2]^

The accurate diagnosis of gingival enlargement poses considerable challenges due to its association with multiple oral and systemic diseases. The etiology of gingival enlargement is multifactorial. Gingival enlargement is an increase in gingival size and a recognized periodontal condition. Based on etiologic factors and pathologic changes, it is classified into:

1. Inflammatory (acute or chronic).
2. Drug-induced (e.g., anticonvulsants: phenytoin; calcium-channel blockers: verapamil/nifedipine; and immunosuppressants: cyclosporine).
3. Systemic associated: I. Hormonal and conditioned: pregnancy, puberty, vitamin C deficiency, plasma cell gingivitis, nonspecific conditioned enlargement (granuloma pyogenicum), II. Systemic diseases: leukemia, granulomatous disorders (sarcoidosis, Crohn disease, Wegener).
4. Neoplastic (benign or malignant gingival tumors).
5. False enlargements (apparent enlargement without true gingival hypertrophy).^[3]^

In leukemia, particularly AML, the gingival tissues are among the oral structures that are predominantly affected.^[4]^ Dentists, being among the first healthcare professionals to encounter such manifestations, play a crucial role in early detection. To our knowledge, this is among the few reports where AML presented solely with gingival enlargement despite an initially normal hematological profile, resulting in diagnostic delay.This report aims to provide dental professionals with a reference to enhance clinical management and reduce the risk of misdiagnosis.

## 2. Case presentation

### 2.1. Diagnosis and etiology

**Figure 1.**
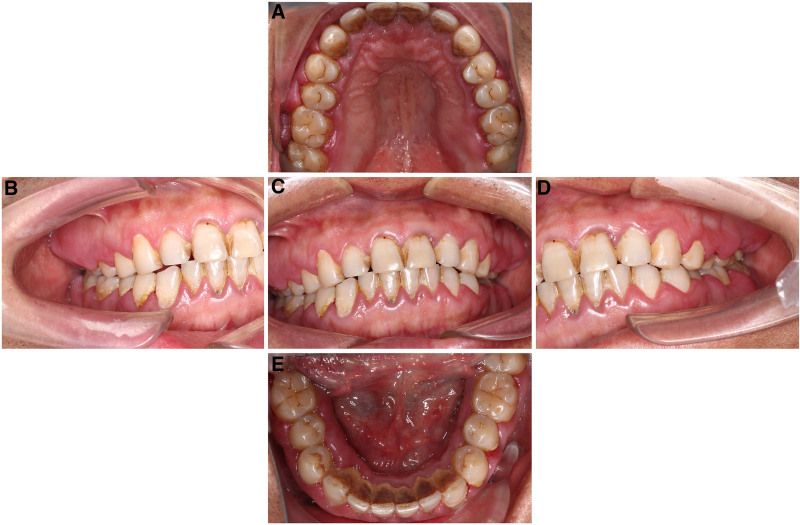
Initial intraoral clinical photographs obtained during the patient’s first visit. The gingival papillae of the lower anterior teeth exhibited signs of redness and swelling, and gingival enlargement was observed in the molar regions. (A) Upper occlusal view; (B) right side view; (C) inter-occlusal view; (D) left side view; and (E) mandibular occlusal view.

**Figure 2.**
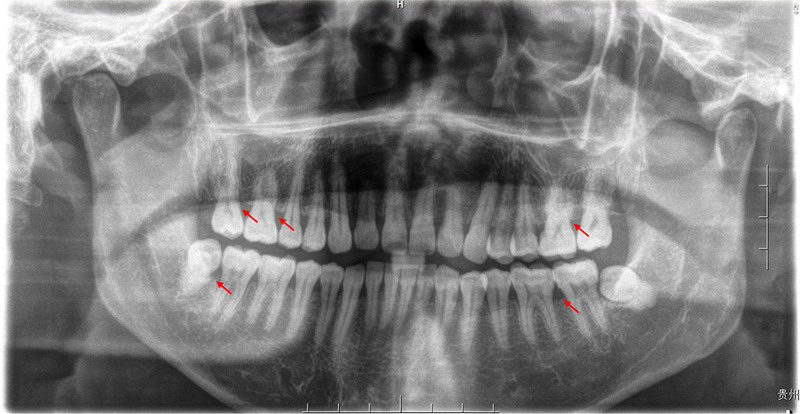
Panoramic X-ray of the patient at the initial visit. Full-mouth radiographs showing mild periodontal bone loss around the premolars and molars (red arrows).

A 45-year-old man was referred to the periodontology department with a chief complaint of gingival bleeding, swelling, and occlusal discomfort persisting for 3 months. One month prior, he had undergone a “flushing” treatment at another hospital; however, the symptoms remained unresolved. At the time of presentation, the hematological analysis revealed no discernible abnormalities (Table 1). The patient reported no recent fever, fatigue, or weight loss. He had a history of hepatitis B and had undergone nasal polyp removal 5 years prior. His physical examination was unremarkable. The patient reported smoking 1 to 2 packs of cigarettes per day for 20 years and had not attempted to quit. Oral examination revealed suboptimal oral hygiene. A minimal amount of tooth surface pigmentation was observed, along with gingival congestion and redness, particularly in the molar region. Gingival stippling had disappeared, with a rounded shape and smooth surface. The gingival papillae of the lower anterior teeth exhibited signs of swelling, and gingival hyperplasia was observed in the molar region, extending to more than two-thirds of the maxillary teeth and more than one-third of the mandibular teeth. Subgingival tartar and attachment loss could be detected by probing to a depth of 4 to 8 mm. The patient exhibited bleeding on probing, but no significant tooth loosening was observed. The remainder of the oral mucosa and general skin exhibited no abnormalities (Fig. 1). Radiographic analysis revealed signs of mild resorption of the alveolar bone and the presence of 2 unerupted third molars (Fig. 2). Based on the above factors (sex, age, no relevant medication history, no abnormal blood counts, and no typical systemic symptoms), we have excluded the diagnoses corresponding to B, C, D, and E mentioned earlier. Chronic inflammatory gingival hyperplasia was the primary provisional diagnosis, given the patient’s poor oral hygiene, significant plaque accumulation, and subgingival calculus.

**Table 1:** Hematological parameters of the patient 1 month before and 1 month after receiving periodontal treatment.

	1 month prior to periodontal treatment	1 month after periodontal treatment	Normal range
Neutrophils	65.5	4.5	40%–75%
Lymphocytes	26.1	57.4	20%–50%
Monocytes	5.6	37.7	3%–10%
Erythrocyte count	5.68	4.05	4.3–5.8 × 10^12^/L
Hematocrit	50.2	36	40%–50%
Platelet count	193	68	125–350 × 10^9^/L
Platelet distribution width	17.1	11.9	9.9–16.1 fL

### 2.2. Treatment objectives

1. Eliminate local irritants and control inflammation through comprehensive periodontal therapy.
2. Improve oral hygiene status and establish effective plaque control habits.
3. Determine the etiology of gingival enlargement and exclude/confirm systemic diseases.
4. Develop targeted treatment plans based on final diagnosis.

### 2.3. Treatment alternatives

1. *Periodontal foundational therapy*: Including full-mouth supragingival scaling, subgingival scaling, and root planing.
2. *Oral hygiene instruction*: Educating patients on proper self-performed plaque control methods.
3. *Close follow-up monitoring*: Regular assessment of periodontal tissue response to treatment.
4. *Multidisciplinary consultation when necessary*: Prompt referral to relevant departments if conventional therapy proves ineffective.

### 2.4. Treatment progress

**Figure 3.**
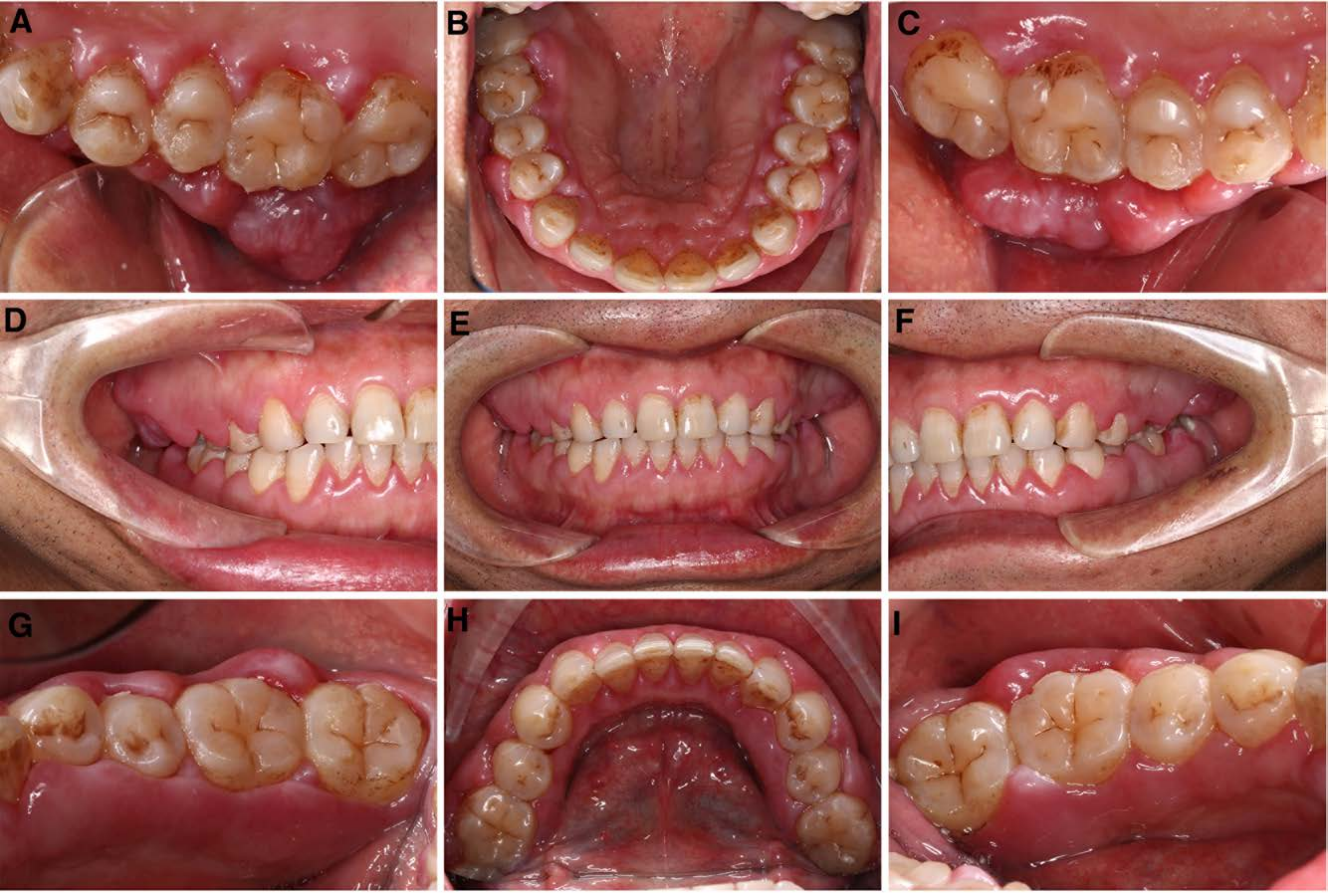
Intraoral photographs at 12 days post-initial periodontal therapy. The gingival surface exhibits a glossy appearance. Significant gingival enlargement is observed in the molar regions of both the maxilla and mandible, with buccal gingiva extending close to the occlusal surface (A, C, G, I). Particularly in the maxillary molar region, the buccal gingiva presents an irregular lobulated morphology (A, C), and locally, the hyperplastic gingiva extends beyond the buccal-lingual diameter of the molars (A). (A) Maxillary right posterior view; (B) upper occlusal view; (C) maxillary left posterior view; (D) right side view; (E) inter-occlusal view; (F) left side view; (G) mandibular right posterior view; (H) mandibular occlusal view; and (I) mandibular right posterior view.

**Figure 4.**
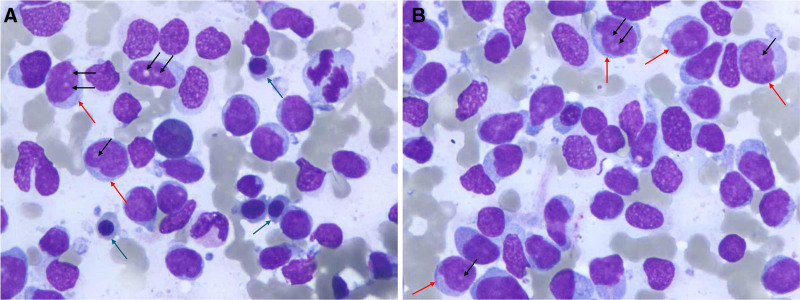
Images from the smears of bone marrow. The images show that the bone marrow is abnormal with acute myeloblastic leukemia. Bone marrow smear, Wright and Giemsa stain, ×1000 (A, B) (the blue arrow points to orthochromatic normoblast, the black arrows point to nucleoli, and the red arrows point to lymphoblasts).

Initial phase included full-mouth supragingival scaling with oral hygiene advice, followed by subgingival scaling and root planing 1 week later. At 3-week follow-up (approximately 21 days after initial presentation), despite improved oral hygiene, gingival enlargement showed rapid progression (Fig. 3). The gingival enlargement demonstrated alarming rapid progression. This marked clinical deterioration, which was entirely disproportionate to the achieved level of local plaque control, raised a high index of suspicion for an underlying systemic malignancy. Consequently, despite the initially normal complete blood count, the patient was immediately referred for further hematological investigation. A repeated blood test was performed (at approximately day 30 post-initial visit), which revealed thrombocytopenia (68 × 10^9^/L) (Table 1). Bone marrow aspiration demonstrated hypercellular marrow with 81% blasts (Fig. 4), and flow cytometry confirmed AML immunophenotype (CD13+, CD33+, CD34+, and CD117+). The patient received “7 + 3” induction chemotherapy with cytarabine and anthracycline.

### 2.5. Treatment results

**Figure 5.**
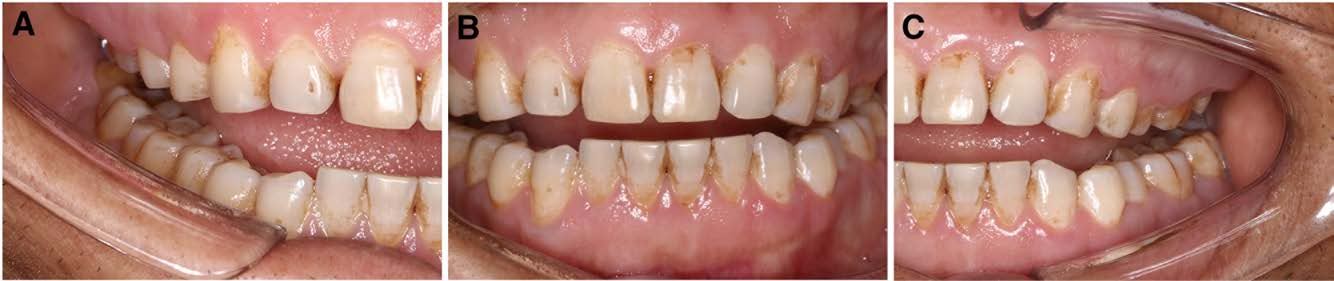
Intraoral photographs obtained at 1-month post-chemotherapy. Following 1 month of treatment, the patient exhibited a significant reduction in gingival swelling. Additionally, slight paleness was observed in the gingiva. (A) Inter-occlusal view; (B) right side view; and (C) left side view.

After 1 month of treatment, the patient exhibited a significant reduction in gingival swelling. Additionally, the gingiva appeared slightly pale (Fig. 5). Eventually, the patient passed away 5 months after the diagnosis.

To clearly illustrate the diagnostic and therapeutic pathway of this complex case, we have summarized the key procedures and decision-making timepoints (Table 2), systematically demonstrating the shift in clinical reasoning from conventional periodontal treatment to the final diagnosis of AML.

**Table 2:** Timeline of key clinical events.

Time point	Key clinical manifestations and examination findings	Clinical decisions and interventions
Prior external care	Gingival bleeding, swelling for 2 months.	“Flushing” treatment
	Generalized gingival overgrowth, bleeding on probing.	
	Complete blood count: normal.	
Initial visit	The gingival enlargement issue has not been resolved.	1. Full-mouth supragingival scaling.
		2. Oral hygiene instruction.
1-week follow-up	Improved oral hygiene.	Complete full-mouth subgingival scaling and root planing.
3-week follow-up	Rapid progression of hyperplasia; Shiny, lobulated appearance; Disproportionate to plaque control.	1. High suspicion for systemic etiology.
		2. Biopsy deferred.
		3. Immediate hematology referral.
Post-referral	Thrombocytopenia (68 × 10^9^/L); Bone marrow: 81% blasts; Flow cytometry: acute myeloid leukemia immunophenotype.	1. Acute myeloid leukemia diagnosis confirmed.
		2. “7 + 3” induction chemotherapy initiated.
1 month post-chemotherapy	Significant regression of gingival hyperplasia.	Supportive oral care.
5 months post-diagnosis	The patient succumbed to the disease.	Not applicable.

## 3. Discussion

Although AML is most often sporadic, it has been established that smoking, a high body mass index, and occupational exposure to benzene and formaldehyde are major risk factors for AML.^[5]^ The patient’s significant 20-pack-year smoking history is a noteworthy aspect of his profile, which may have contributed to the development of the AML.^[6,7]^ Individuals afflicted with hematological disorders frequently manifest systemic symptoms, including weight loss, fever, and malaise. These symptoms are predominantly linked to anemia, neutropenia, and thrombocytopenia.^[8–10]^ Over 30% of individuals diagnosed with leukemia experience various oral manifestations.^[11]^ Given the abundance of capillaries in gingival tissues, this region is extremely susceptible to leukemia-induced oral lesions. About 36% of patients present with gingival overgrowth.^[12,13]^ The current classification and standard treatment paradigms for AML are well-established, ranging from induction chemotherapy to transplantation, yet long-term survival remains poor.^[14–19]^ This context of aggressive disease and challenging management underscores the critical importance of early diagnosis.

The current understanding of the relationship between periodontal and systemic diseases has significantly advanced. Notably, periodontal conditions directly associated with hematological disorders, such as leukemia, are now recognized as manifestations of systemic diseases.^[20,21]^ Gingival enlargement commonly results from chronic inflammation, primarily because of prolonged plaque accumulation. In contrast, the severity and extent of the gingival changes and the duration of gingival enlargement described in the present case are inconsistent. This may be because of direct infiltration of immature leukocytes (primitive cells) or neutropenia-related mechanisms accompanied by secondary thrombocytopenia and immunodeficiency.^[12,22,23]^ This pathological infiltration leads to gingival structural changes manifested by tissue thickening and pseudo-pocket formation. Subsequently, this triggers an ongoing inflammatory cascade response through secondary microbial colonization.^[22]^

Our review identified 8 documented AML cases where oral manifestations (mainly gingival enlargement or bleeding)^[12,22,24–29]^ were the presenting feature, with most cases showing concurrent systemic symptoms and complete blood count abnormalities (Table 3). The uniqueness of this case lies in the patient’s completely normal hematological parameters at the initial diagnosis, which posed a significant diagnostic challenge. The clinical presentation of gingival overgrowth—characterized by rapid progression, a shiny and lobulated appearance, and lack of response to conventional periodontal therapy—served as a critical clue suggesting an underlying systemic malignancy.

**Table 3:** Summary of acute myeloid leukemia cases with gingival manifestations.

Case	Age	Sex	Gingival findings	Systemic findings	Posttreatment gingival status	Patient outcome	References
1	77	Male	No overgrowth; spontaneous oozing around #14, 15, large clot	Mild hypertension; no other notable symptoms	Not described	Died months post-diagnosis	^[24]^
2	32	Male	Generalized bluish-purple overgrowth, floor of mouth ecchymosis; soft, edematous	Dyspnea, enterocolitis history, weight loss, hepatomegaly, anemia	Not described	Died 4 days post-diagnosis (pre-chemotheraphy)	^[25]^
3	43	Female	Generalized inflamed erythematous overgrowth, no stippling; firm, tender, bleeds easily	Initially asymptomatic; later developed lymphadenopathy, hepatosplenomegaly	No periodontal therapy	Poor chemo response, died within 10 days of diagnosis	^[26]^
4	49	Male	No overgrowth; spontaneous bleeding palatal to upper incisors, 2-year duration	Exertional breathlessness, severe fatigue, pallor, koilonychias	Not described	Complete remission after induction chemo, planned stem cell transplant	^[27]^
5	30	Male	Severe overgrowth covering crowns, hemorrhagic, ulcerated, reddish-purple	Fatigue, weight loss, fever, tender submandibular lymph nodes	Periodontal therapy postponed	Completed 3 chemo courses, on follow-up	^[12]^
6	10	Female	Generalized, firm, pink to dark purple overgrowth, spontaneous bleeding	Lymphadenopathy, rapid weight loss, fever, skin petechiae	Significant resolution post-chemotheraphy (3 wk)	Completed chemo, in remission (12 mo)	^[22]^
7	11	Female	Generalized pale, bulbous overgrowth, loss of stippling, focal hemorrhage	Mild fever, sore throat, fatigue, weight loss, tender lymph nodes	Near-normal appearance post-chemotheraphy (7 wk)	Febrile neutropenia managed, stable, consolidation chemo	^[28]^
8	26	Male	Diffuse reddish swelling, palpable masses, bleeding on probing, pseudopockets	No systemic symptoms other than oral findings	Not explicitly described	Not explicitly described	^[29]^

As seen from the cases in Table 3, systemic malignancies can precede detectable changes in peripheral blood. Our case further demonstrated a dynamic progression from normal complete blood counts to overt thrombocytopenia within 2 months, highlighting the necessity for serial monitoring (Table 1). Pathophysiologically, this enlargement stems from direct infiltration of gingival connective tissue by leukemic blasts, which disrupts the normal gingival architecture and triggers a secondary inflammatory response, creating a vicious cycle of enlargement and pseudo-pocket formation. The resolution of enlargement following induction chemotherapy, as observed in our case and by Yang et al,^[30]^ further confirms its direct association with the leukemic process.

In terms of diagnostic strategy, our case contrasts with the approach described by Fernandes et al^[22]^ and Zisis et al.^[29]^ In the course of their study, patients exhibited systemic symptoms and abnormal blood cell counts, thus making gingival biopsy a critical step for confirming leukemic infiltration and performing immunophenotyping. In contrast, given the patient’s initially normal hematological parameters, performing a gingival biopsy would not only carry a significant risk of bleeding but also offer limited diagnostic value. Therefore, we bypassed this invasive procedure and proceeded directly to bone marrow examination, the diagnostic gold standard. This decision reflects the principle of prioritizing the most diagnostically valuable and least risky method when systemic malignancy is suspected.

Based on this case and literature review, we summarize the following “red flags” that should raise suspicion of an underlying systemic malignancy like leukemia:

*Refractoriness to conventional therapy*: Gingival enlargement and bleeding persist or worsen despite professional plaque removal and optimal oral hygiene.

*Disproportionate clinical features*: The severity and appearance (e.g., lobulated, nodular) of the enlargement are far greater than expected from the level of local irritants.

*Atypical bleeding*: Spontaneous bleeding or excessive bleeding on probing that is inconsistent with the degree of inflammation.

*Rapid progression*: Noticeable enlargement of gingival tissues over a period of weeks.

*Presence of systemic symptoms*: Unexplained fever, fatigue, weight loss, night sweats, or easy bruising.

*Actionable recommendations*: Upon recognizing any of these signs, especially in combination, dental clinicians should:

1. Meticulously document the history and oral findings.
2. Initiate immediate referral to a hematologist or physician for consultation.
3. Request a complete blood count with peripheral smear as a first-line investigation.
4. Do not delay referral awaiting abnormal blood results; a normal complete blood count does not rule out leukemia.
5. Defer any non-emergent invasive dental procedures until hematological evaluation is complete.
6. For patients diagnosed with atypical gingival overgrowth, following the completion of fundamental periodontal treatment, the implementation of a stringent short-term follow-up protocol is imperative. This protocol should mandate the conduction of mandatory follow-up examinations within a timeframe of 2 weeks.

Periodontal and oral care for patients with leukemia must be administered within the framework of a multidisciplinary collaboration between hematology and periodontics.^[30]^ It is imperative that patients undergo dental screening and oral hygiene advice before receiving induction chemotherapy, which can prevent local and systemic complications. The goal of treatment is to eliminate infection and address acute or potential problems. For patients with severe pancytopenia or platelet counts <20 × 10^9^/L, careful toothbrushing is advised to prevent gingival bleeding. If that is not feasible, wet cotton soaked with high-fluoride toothpaste can be used instead.^[31]^ Clinical dental screening before induction chemotherapy in patients with AML resulted in a 6-fold reduction in infectious dental emergencies during induction chemotherapy.^[32]^ The clinical acceptance of invasive therapy is contingent upon a combination of platelet and neutrophil counts, with a platelet count ≥50 × 10^9^/L and a neutrophil count ≥1.0 × 10^9^/L identified as commonly accepted safe thresholds. For patients with low platelet counts, treatment options include posttransfusion management and local hemostasis.^[11]^ High prevalence of gingivitis/periodontitis has been identified as an independent risk factor for infectious complications during chemotherapy.^[33]^ When systemic comorbidities are adequately controlled, periodontal debridement (scaling,^[30]^ root planing, and curettage) may be performed under prophylactic antibiotic coverage. Periodontal surgery should be avoided until remission, followed by twice-daily oral rinsing with 0.1% to 0.2% chlorhexidine gluconate to minimize oral complications during remission–induction chemotherapy. Dentists are an important part of the multidisciplinary team during the pre- and posttreatment periods in AML.^[13]^ Gingival enlargement often resolves following successful chemotherapy for leukemia.^[34]^ The patient in the present case received basic periodontal treatment and chemotherapy, resulting in the resolution of most of the gingival swelling following local plaque control. In the context of post-remission supportive care, several adjunctive therapies—such as ozone therapy,^[35]^ probiotics,^[36]^ and photobiomodulation^[37]^—may play a role in managing localized oral inflammation and improving periodontal health. However, their application in the setting of leukemia-related oral complications warrants further investigation.

The patient initially perceived his condition as a localized dental issue. The rapid deterioration despite treatment caused significant anxiety, and the eventual leukemia diagnosis was devastating. The present report is subject to certain limitations. The most significant issue is the absence of long-term follow-up data due to the patient’s poor outcome, which resulted in their demise 5 months post-diagnosis. Consequently, the present study is unable to report on long-term survival or the potential effects of modernized AML therapies (e.g., targeted agents, transplantation) on the oral manifestations. However, this unfortunate outcome itself underscores the aggressiveness of the disease and the paramount importance of early detection and intervention.

## 4. Conclusion

In conclusion, while gingival manifestations of AML are well-documented, this report particularly emphasizes the diagnostic value of persistent gingival enlargement unresponsive to conventional periodontal therapy as a critical indicator of potential systemic malignancy. Our case underscores the imperative for dental practitioners to maintain heightened clinical suspicion even when initial hematological parameters appear normal. The recognition of key red flags—including rapid progression, disproportionate gingival changes, and refractoriness to standard treatment—should prompt immediate hematological referral regardless of preliminary laboratory findings. Through vigilant assessment of these warning signs, dental professionals can substantially reduce diagnostic delays and play a decisive role in early detection of systemic malignancies, ultimately improving patient prognosis and clinical outcomes.

## Acknowledgments

We thank the patient and his family for the publication of this study.

## Author contributions

**Data curation:** Wenxiu Li, Liang Luo.

**Funding acquisition:** Wenxiu Li, Liang Luo, Dandan Wu.

**Supervision:** Fugen Ding.

**Writing – original draft:** Dandan Wu.

**Writing – review & editing:** Fugen Ding.

## References

[1] Pollyea DA Bixby D Perl A. NCCN guidelines insights: acute myeloid leukemia, version 2.2021. J Natl Compr Canc Netw. 2021;19:16–27. 33406488 10.6004/jnccn.2021.0002

[2] Foreman KJ Marquez N Dolgert A. Forecasting life expectancy, years of life lost, and all-cause and cause-specific mortality for 250 causes of death: reference and alternative scenarios for 2016-40 for 195 countries and territories. Lancet. 2018;392:2052–90. 30340847 10.1016/S0140-6736(18)31694-5 PMC6227505

[3] Beaumont J Chesterman J Kellett M Durey K. Gingival overgrowth: Part 1: aetiology and clinical diagnosis. Br Dent J. 2017;222:85–91. 28127024 10.1038/sj.bdj.2017.71

[4] Quispe RA Aguiar EM de Oliveira CT Neves ACX Santos PSDS. Oral manifestations of leukemia as part of early diagnosis. Hematol Transfus Cell Ther. 2022;44:392–401. 34862157 10.1016/j.htct.2021.08.006 PMC9477758

[5] Yi M Li A Zhou L Chu Q Song Y Wu K. The global burden and attributable risk factor analysis of acute myeloid leukemia in 195 countries and territories from 1990 to 2017: estimates based on the global burden of disease study 2017. J Hematol Oncol. 2020;13:72. 32513227 10.1186/s13045-020-00908-z PMC7282046

[6] Colamesta V D’Aguanno S Breccia M Bruffa S Cartoni C La Torre G. Do the smoking intensity and duration, the years since quitting, the methodological quality and the year of publication of the studies affect the results of the meta-analysis on cigarette smoking and acute myeloid leukemia (AML) in adults? Crit Rev Oncol Hematol. 2016;99:376–88. 26830008 10.1016/j.critrevonc.2016.01.003

[7] Lu X Peng X Zhang Y. Global and Chinese trends in acute myeloid leukemia burden (1990–2021): a comprehensive analysis based on the GBD study. Front Med (Lausanne). 2025;12:1629111. 40978755 10.3389/fmed.2025.1629111 PMC12446328

[8] DiNardo CD Erba HP Freeman SD Wei AH. Acute myeloid leukaemia. Lancet. 2023;401:2073–86. 37068505 10.1016/S0140-6736(23)00108-3

[9] Hochhaus A Saussele S Rosti G. Chronic myeloid leukaemia: ESMO clinical practice guidelines for diagnosis, treatment and follow-up. Ann Oncol. 2018;29:iv261. 30285223 10.1093/annonc/mdy159

[10] Malard F Mohty M. Acute lymphoblastic leukaemia. Lancet. 2020;395:1146–62. 32247396 10.1016/S0140-6736(19)33018-1

[11] Watson E Wood RE Maxymiw WG Schimmer AD. Prevalence of oral lesions in and dental needs of patients with newly diagnosed acute leukemia. J Am Dent Assoc. 2018;149:470–80. 29606275 10.1016/j.adaj.2018.01.019

[12] Misirlioglu M Adisen MZ Yilmaz S. Diagnosis of acute myeloid leukemia in a dental hospital; report of a case with severe gingival hypertrophy. Niger J Clin Pract. 2015;18:573–6. 25966736 10.4103/1119-3077.151803

[13] Cammarata-Scalisi F Girardi K Strocchio L. Oral manifestations and complications in childhood acute myeloid leukemia. Cancers (Basel). 2020;12:1634. 32575613 10.3390/cancers12061634 PMC7352340

[14] Zhang Z Huang J Zhang Z. Application of omics in the diagnosis, prognosis, and treatment of acute myeloid leukemia. Biomark Res. 2024;12:60. 38858750 10.1186/s40364-024-00600-1 PMC11165883

[15] Guijarro F Garrote M Villamor N Colomer D Esteve J López-Guerra M. Novel tools for diagnosis and monitoring of AML. Curr Oncol. 2023;30:5201–13. 37366878 10.3390/curroncol30060395 PMC10297692

[16] Doehner H Wei AH Pocock C. The QUAZAR AML-001 maintenance trial: results of a phase III international, randomized, double-blind, placebo-controlled study of oral CC-486 in patients with acute myeloid leukemia (AML) in first remission. Blood. 2019;134:LBA-3.

[17] Kantarjian HM DiNardo CD Kadia TM. Acute myeloid leukemia management and research in 2025. CA Cancer J Clin. 2025;75:46–67. 39656142 10.3322/caac.21873 PMC11745214

[18] Albinger N Pfeifer R Nitsche M. Primary CD33-targeting CAR-NK cells for the treatment of acute myeloid leukemia. Blood Cancer J. 2022;12:61. 35418180 10.1038/s41408-022-00660-2 PMC9007937

[19] Sasaki K Ravandi F Kadia TM. De novo acute myeloid leukemia: a population-based study of outcome in the United States based on the surveillance, epidemiology, and end results (SEER) database, 1980 to 2017. Cancer. 2021;127:2049–61. 33818756 10.1002/cncr.33458 PMC11826308

[20] Caton JG Armitage G Berglundh T. A new classification scheme for periodontal and peri-implant diseases and conditions – introduction and key changes from the 1999 classification. J Clin Periodontol. 2018;45:S1–8. 29926489 10.1111/jcpe.12935

[21] Łobacz M Mertowska P Mertowski S. The bloody crossroads: interactions between periodontitis and hematologic diseases. Int J Mol Sci. 2024;25:6115. 38892299 10.3390/ijms25116115 PMC11173219

[22] Fernandes KS Gallottini M Castro T Amato MF Lago JS Braz-Silva PH. Gingival leukemic infiltration as the first manifestation of acute myeloid leukemia. Spec Care Dentist. 2018;38:160–2. 29645289 10.1111/scd.12283

[23] George N Santhosh VC Kumar H Gopal S. Gingival enlargement in myelodysplastic syndrome. J Indian Soc Periodontol. 2015;19:687–9. 26941522 10.4103/0972-124X.164761 PMC4753716

[24] Gleeson P. Spontaneous gingival haemorrhage: case report. Aust Dent J. 2002;47:174–5. 12139274 10.1111/j.1834-7819.2002.tb00324.x

[25] Reenesh M Munishwar S Rath SK. Generalised leukaemic gingival enlargement: a case report. J Oral Maxillofac Res. 2012;3:e5. 10.5037/jomr.2012.3305 PMC3886083 24422017

[26] Babu SP Kashyap V Sivaranjani P Agila S. An undiagnosed case of acute myeloid leukemia. J Indian Soc Periodontol. 2014;18:95–7. 24744555 10.4103/0972-124X.128257 PMC3988656

[27] Guan G Firth N. Oral manifestations as an early clinical sign of acute myeloid leukaemia: a case report. Aust Dent J. 2015;60:123–7. 25721286 10.1111/adj.12272

[28] Sharan J Mohapatra S Chhabra G. Gingival hyperplasia: an initial oral manifestation of acute myeloid leukemia. J Indian Soc Periodontol. 2023;27:201–6. 37152465 10.4103/jisp.jisp_54_22 PMC10159087

[29] Zisis V Zisis S Anagnostou E Dabarakis N Poulopoulos A Andreadis D. Gingival enlargement can constitute the only diagnostic sign of leukemia: report of an unusual case. Cureus. 2023;15:e47959. 38034185 10.7759/cureus.47959 PMC10685703

[30] Yang G Tu Y Hu W Feng X. 10-year follow-up of adolescent leukemia diagnosed through gingiva: a case report [published online ahead of print February 14, 2025]. Clin Adv Periodontics. doi: 10.1002/cap.10340. 10.1002/cap.10340 39949297

[31] Abed H Alhabshi M Alkhayal Z Burke M Nizarali N. Oral and dental management of people with myelodysplastic syndromes and acute myeloid leukemia: a systematic search and evidence-based clinical guidance. Spec Care Dentist. 2019;39:406–20. 31087570 10.1111/scd.12384

[32] Watson EE Metcalfe JE Kreher MR Maxymiw WG Glogauer M Schimmer AD. Screening for dental infections achieves 6-fold reduction in dental emergencies during induction chemotherapy for acute myeloid leukemia. JCO Oncol Pract. 2020;16:e1397–405. 32609586 10.1200/OP.20.00107

[33] Allareddy V Prakasam S Martinez-Schlurmann N. Poor oral health linked with increased risk of infectious complications in adults with leukemia. J Mass Dent Soc. 2015;64:38–42. 26727815

[34] Cooper CL Loewen R Shore T. Gingival hyperplasia complicating acute myelomonocytic leukemia. J Can Dent Assoc. 2000;66:78–9. 10730004

[35] Colombo M Gallo S Garofoli A Poggio C Arciola CR Scribante A. Ozone gel in chronic periodontal disease: a randomized clinical trial on the anti-inflammatory effects of ozone application. Biology (Basel). 2021;10:625. 34356480 10.3390/biology10070625 PMC8301177

[36] Meng N Liu Q Dong Q Gu J Yang Y. Effects of probiotics on preventing caries in preschool children: a systematic review and meta-analysis. J Clin Pediatr Dent. 2023;47:85–100. 36890746 10.22514/jocpd.2023.014

[37] Scribante A Gallo S Pascadopoli M. Management of periodontal disease with adjunctive therapy with ozone and photobiomodulation (PBM): a randomized clinical trial. Photonics. 2022;9:138.

